# Development and validation of a prognostic nomogram for predicting cancer-specific survival in patients with metastatic clear cell renal carcinoma: A study based on SEER database

**DOI:** 10.3389/fonc.2022.949058

**Published:** 2022-09-28

**Authors:** Guangyi Huang, Jie Liao, Songwang Cai, Zheng Chen, Xiaoping Qin, Longhong Ba, Jingmin Rao, Weimin Zhong, Ying Lin, Yuying Liang, Liwei Wei, Jinhua Li, Kaifeng Deng, Xiangyue Li, Zexiong Guo, Liang Wang, Yumin Zhuo

**Affiliations:** ^1^ Department of Urology, The First Affiliated Hospital of Jinan University, Jinan University, Guangzhou, China; ^2^ Department of Oncology, The First Affiliated Hospital of Jinan University, Jinan University, Guangzhou, China; ^3^ Department of Thoracic Surgery, The First Affiliated Hospital of Jinan University, Guangzhou, China

**Keywords:** metastatic clear cell renal cell carcinoma, Surveillance, Epidemiology, and End Results (SEER), nomogram, prognosis, survival analysis

## Abstract

**Objectives:**

Clear cell renal cell carcinoma (ccRCC) is highly prevalent, prone to metastasis, and has a poor prognosis after metastasis. Therefore, this study aimed to develop a prognostic model to predict the individualized prognosis of patients with metastatic clear cell renal cell carcinoma (mccRCC).

**Patients and Methods:**

Data of 1790 patients with mccRCC, registered from 2010 to 2015, were extracted from the Surveillance, Epidemiology and End Results (SEER) database. The included patients were randomly divided into a training set (n = 1253) and a validation set (n = 537) based on the ratio of 7:3. The univariate and multivariate Cox regression analyses were used to identify the important independent prognostic factors. A nomogram was then constructed to predict cancer specific survival (CSS). The performance of the nomogram was internally validated by using the concordance index (C-index), calibration plots, receiver operating characteristic curves, net reclassification improvement (NRI), integrated discrimination improvement (IDI), and decision curve analysis (DCA). We compared the nomogram with the TNM staging system. Kaplan–Meier survival analysis was applied to validate the application of the risk stratification system.

**Results:**

Diagnostic age, T-stage, N-stage, bone metastases, brain metastases, liver metastases, lung metastases, chemotherapy, radiotherapy, surgery, and histological grade were identified as independent predictors of CSS. The C-index of training and validation sets are 0.707 and 0.650 respectively. In the training set, the AUC of CSS predicted by nomogram in patients with mccRCC at 1-, 3- and 5-years were 0.770, 0.758, and 0.757, respectively. And that in the validation set were 0.717, 0.700, and 0.700 respectively. Calibration plots also showed great prediction accuracy. Compared with the TNM staging system, NRI and IDI results showed that the predictive ability of the nomogram was greatly improved, and DCA showed that patients obtained clinical benefits. The risk stratification system can significantly distinguish the patients with different survival risks.

**Conclusion:**

In this study, we developed and validated a nomogram to predict the CSS rate in patients with mccRCC. It showed consistent reliability and clinical applicability. Nomogram may assist clinicians in evaluating the risk factors of patients and formulating an optimal individualized treatment strategy.

## Introduction

Renal cell carcinoma (RCC), referred to as renal cancer, is one of the most common malignant tumors of the urinary system (1). According to the latest statistics of the World Health Organization, there were 431,288 new cases of RCC worldwide in 2020, with an incidence rate of 2.2%, including 179,368 deaths and a mortality rate of 1.8%. In addition, there were 271,249 new male cases and 160,039 new female cases. The male incidence rate was 1.7 times higher than the women. RCC accounts for about 90% of all renal malignancies. The main pathological types include clear cell renal cell carcinoma (ccRCC), papillary carcinoma, and chromophobe cell carcinoma. ccRCC is the most common pathological subtype, accounting for about 70% of all RCC (2, 3).

Previous studies have reported that about 18%-30% of patients with RCC have systemic metastasis at the initial diagnosis, and about one third of patients develop metastatic RCC (mRCC) during long-term follow-up after radical nephrectomy (4, 5). Generally, patients with mRCC have a poor prognosis, with a median overall survival of about 13 months (6). Among clear cell carcinoma, papillary carcinoma, and chromophobe cell carcinoma, ccRCC has the most patients, the worst prognosis, and the most prone to distant metastasis (7, 8). The lung is considered to be the most common metastatic site in patients with ccRCC, followed by bone. The ccRCC can also be transferred to liver, brain, adrenal gland (9, 10). Although with the development of targeted drugs and immunotherapy, the 5-year survival rate of patients with mccRCC has increased from 7.3% to 12.3% (8, 11), the prognosis is still poor. As a consequence, it is necessary to study the prognostic factors of patients with mccRCC and establish a prediction model.

The traditional treatment decision-making and prognosis evaluation of malignant tumors are mainly determined according to the American Joint Committee on Cancer (AJCC) TNM staging system. It only considers the AJCC clinical staging obtained by TNM and does not consider the factors related to the prognosis of patients, such as age, surgery, radiotherapy, transfer site, number of transferred organs, histological grade and so on. The TNM staging system of AJCC is also the most commonly used prognostic evaluation system for RCC (12). Nevertheless, in clinical practice, significant survival differences were observed in patients with mccRCC with the same TNM stage. Therefore, another prognostic tool is needed to better predict the prognosis of patients with mccRCC. Nomogram is a graphical representation of the multivariate model. Compared to the traditional TNM staging system, nomogram can integrate more prognostic factors and is more accurate in predicting the survival of patients with some malignant tumors (13–16). Nomogram is also a visible and reliable statistical prediction tool. It is widely used to provide tailored individual prognosis information. The nomogram consists of basic variables such as demographics, tumor characteristics, and treatment characteristics (17). In the past few years, several prognostic nomograms have been developed for RCC patients (18), some of which are based only on data from patients with mRCC. However, for the nomogram of distant metastasis of ccRCC, we only found a prognostic nomogram of lung metastasis of ccRCC (18). The nomogram of prognosis of ccRCC patients with metastasis of other organs or multiple organs has not been found yet. There are significant differences in survival among mRCC patients with different pathological types. Therefore, the previous nomogram based on mRCC patient data may show low accuracy when used in patients with mccRCC. It is necessary to establish a prognostic nomogram model of mccRCC patients to improve the prediction accuracy. As we know, this study is the first time to establish the prognostic nomogram model of patients with mccRCC based on SEER database and to compare the nomogram with the TNM staging system using the NRI and IDI. This study aims to provide a more accurate prognostic prediction for patients and provide a reference for clinicians to manage patients.

## Patients and methods

### Data source and patients selection

**Figure 1:**
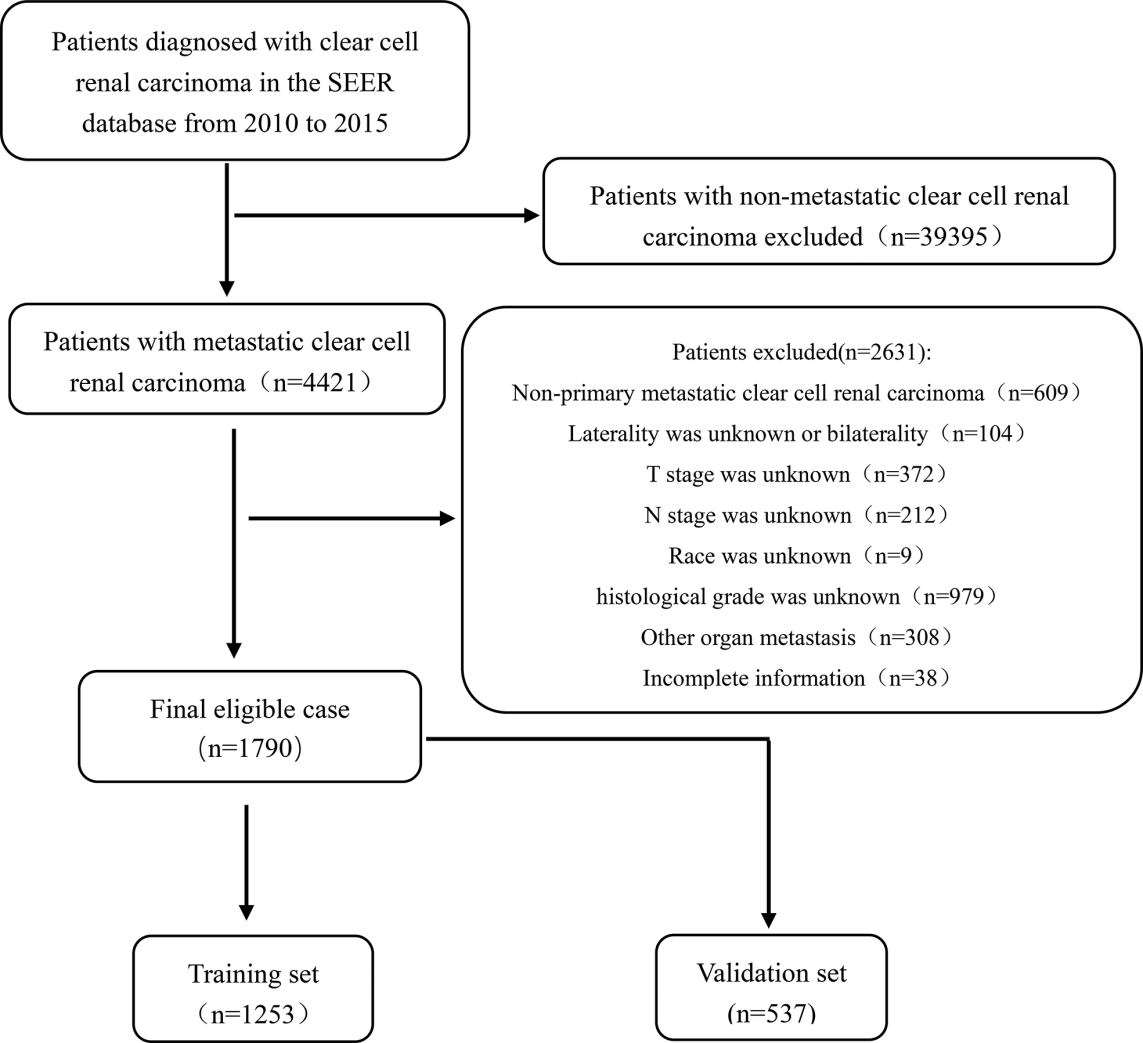
Flowchart showing the selection of patients from the SEER database, based on the inclusion and exclusion criteria outlined above; 1,790 patients were included in this study.

Data of patients diagnosed with mccRCC were collected from the Surveillance, Epidemiology, and End Results (SEER) database (https://seer.cancer.gov/) according to the International Classification of Tumor Diseases Third Edition (ICD-O-3). The inclusion criteria were as follows (1): The first diagnosis was primary clear cell renal cell carcinoma; (2) Distant organ metastasis, including lung, liver, brain, and bone metastases; (3) Unilateral primary clear cell renal cell carcinoma; (4) The age of diagnosis was 18 years or older; (5) The registration information is complete. The exclusion criteria were as follows: (1) Non primary clear cell renal cell carcinoma; (2) Bilateral or lateral unclear primary metastatic clear cell renal cell carcinoma; (3) Other organ metastases (non-lung, non-liver, non-brain, non-bone, and other organ metastasis); (4) Race, T-stage, N-stage or histological grade were unknown; (5) Cases with incomplete information. After screening, 1790 eligible mccRCC patients were finally included in the cohort. The process of data was shown in **Figure 1**. Patients were randomly divided into two sets (training set, n = 1253, and validation set, n = 537) based on the ratio of 7:3. Since SEER is a publicly available database, studies using the SEER database do not require ethical board approval and patient consent.

### Data collection and end point

The variables in this study included diagnostic age, gender, race, laterality, T-stage, N-stage (TNM stage according to the seventh edition of the American Joint Commission on Cancer staging system), lung metastases, liver metastases, brain metastases, bone metastases, chemotherapy, radiotherapy, surgery, histological grade, survival status (survival and death), and survival time. The primary endpoint in this study was Cancer specific survival (CSS), which related to the death of mccRCC. Survival time was calculated from the date of diagnosis to the date of the last follow-up or until the date of death due to cancer and its complications.

### Statistical analysis

The included patients were randomly divided into a training set (n = 1253) and a validation set (n = 537) based on the ratio of 7:3. The Student’s t-test and Chi-square test were performed for continuous and categorical variables, respectively, to explore the baseline characteristics of patients in the two sets. Continuous variables were reported as median with range, and categorical variables as frequencies and proportions. The optimal cutoff values for age were evaluated using the X-tile software. In the training set, the univariate Cox regression analysis was performed to identify the significant prognostic factors. Afterwards, they were incorporated into the multivariable Cox proportional hazards regression model to further determine the relationship between each variable and survival outcomes of patients with mccRCC when their p-value was under 0.05. All results were shown as hazards ratios (HR) and 95% confidence intervals (95%CI). The influencing factors was used to screen in the multivariable Cox regression model, and a nomogram for predicting CSS in 1-, 3- and 5-years was constructed. The predictive discriminative ability of the nomogram was determined by Harrell’s concordance index (C-index) and the receiver operating characteristics (ROC) curves, the area under the curve (AUC). The accuracy of the nomogram in predicting 1 -, 3 - and 5-year CSS was evaluated by Calibration plots. In addition, the net reclassification improvement (NRI) and integrated discrimination improvement (IDI) were used to assess whether the nomogram was more accurate than the AJCC TNM staging system or not. And decision curve analysis (DCA) was used to evaluate the clinical utility of the nomogram.

In addition, we calculated the total score for each patient based on the nomogram. According to the total score, we constructed a risk stratification model, which divided the total cohort into two different risk groups (low-risk group and high-risk group). The best cut-off value was analyzed by X-tile software. The survival differences between low-risk and high-risk groups were assessed by Kaplan Meier survival analysis.

All analyses were performed using the spss27.0 and the statistical package R 4.1.2 (http://www.R-project.org). The two-sided p-value of less than 0.05 was considered statistically significant. All procedures involving human participants in this study met the ethical standards described in the 1964 Declaration of Helsinki and its subsequent amendments.

## Results

### Patients’ baseline characteristics

In total, 1,790 patients with mccRCC were enrolled in this study. 1,253 patients (70%) were distributed into the training set while 537 patients (30%) into the validation set. Baseline demographical and clinical characteristics of the study population are shown in **Table 1**. The majority of patients were male (1261, 70.45%), white (1537, 85.87%), and under 66 years of age (1233, 68.88%). In terms of treatment, most patients underwent surgery (1468, 82.01%), while fewer patients received radiotherapy (556, 31.06%). The mean survival time was 28.41 ± 25.36, 28.80 ± 25.85, and 27.51 ± 24.15 months in the total cohort, training set, and validation set, respectively. The difference between the training and validation sets was not statistically significant in all variables (p > 0.05).

**Table 1:** Baseline demographic and clinical characteristics of patients in the training set and validation set.

characteristics	total (n = 1790)	training set (n = 1253)	validation set (n = 537)	p-value
Total	1790	1253	537	
Survival months	28.41 ± 25.36	28.80 ± 25.85	27.51 ± 24.15	0.323
Age, n (%)				
≤66	1233 (68.88)	858 (68.48)	375 (69.83)	0.570
>66	557 (31.12)	395 (31.52)	162 (30.17)	
Sex, n (%)				
Male	1261 (70.45)	888 (70.87)	373 (69.46)	0.549
Female	529 (29.55)	365 (29.13)	164 (30.54)	
Race, n (%)				
White	1537 (85.87)	1073 (85.63)	464 (86.41)	0.383
Black	103 (5.75)	78 (6.23)	25 (4.66)	
Other	150 (8.38)	102 (8.14)	48 (8.94)	
Laterality, n (%)				
Left	910 (50.84)	618 (49.32)	292 (54.38)	0.050
Right	880 (49.16)	635 (50.68)	245 (45.62)	
T-stage, n (%)				
T1	246 (13.74)	170 (13.57)	76 (14.15)	0.205
T2	327 (18.27)	236 (18.83)	91 (16.95)	
T3	1008 (56.31)	690 (55.07)	318 (59.22)	
T4	209 (11.68)	157 (12.53)	52 (9.68)	
N-stage, n (%)				
N0	1368 (76.42)	966 (77.09)	402 (74.86)	0.307
N1	422 (23.58)	287 (22.91)	135 (25.14)	
Bone metastases, n (%)				
Yes	628 (35.08)	438 (34.96)	190 (35.38)	0.863
No	1162 (64.92)	815 (65.04)	347 (64.62)	
Brain metastases, n (%)				
Yes	219 (12.23)	157 (12.53)	62 (11.55)	0.560
No	1571 (87.77)	1096 (87.47)	475 (88.45)	
Liver metastases, n (%)				
Yes	242 (13.52)	178 (14.21)	64 (11.92)	0.195
No	1548 (86.48)	1075(85.79)	473 (88.08)	
Lung metastases, n (%)				
Yes	1304 (72.85)	913 (72.87)	391 (72.81)	0.981
No	486 (27.15)	340 (27.13)	146 (27.19)	
Chemotherapy, n (%)				
Yes	1037 (57.93)	731 (58.34)	306 (56.98)	0.594
No	753 (42.07)	522 (41.66)	231 (43.02)	
Radiotherapy, n (%)				
Yes	556 (31.06)	383 (30.57)	173 (32.22)	0.490
No	1234 (68.94)	870 (69.43)	364 (67.78)	
Surgery, n (%)				
Yes	1468 (82.01)	1017(81.17)	451 (83.99)	0.155
No	322 (17.99)	236(18.83)	86 (16.01)	
Histological grade, n (%)				
Well differentiated	476 (26.59)	345(27.53)	131 (24.39)	0.289
Moderately differentiated	776 (43.35)	530(42.30)	246 (45.81)	
Poorly differentiated	538 (30.06)	378(30.17)	160 (29.80)	

### Screening for prognostic factors of CSS

**Figure 2:**
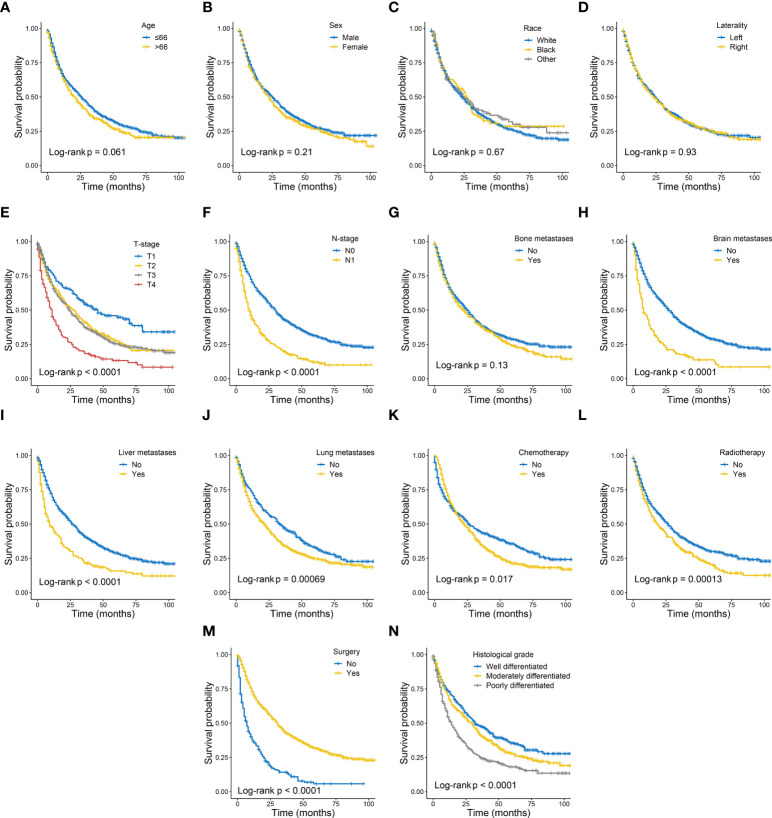
Kaplan–Meier curves of cancer specific survival in patients with metastatic clear cell renal cell carcinoma stratified by age **(A)**, sex **(B)**, race **(C)**, laterality **(D)**, T-stage **(E)**, N-stage **(F)**, bone metastases **(G)**, brain metastases **(H)**, liver metastases **(I)**, lung metastases **(J)**, chemotherapy **(K)**, radiotherapy **(L)**, surgery **(M)** and histological grade **(N)**.

The univariate and multivariate Cox proportional risk regression analyses were performed to demonstrate the association between the selected variables and oncologic outcomes, the univariate and multivariate Cox regression analysis of included variables for CSS in training set are shown in **Table 2**. The univariate Cox regression analysis identified nine variables (T-stage, N-stage, brain metastases, liver metastases, lung metastases, chemotherapy, radiotherapy, surgery, and histological grade) as factors associated with CSS. Although the statistical analysis showed that age and bone metastases were not statistically significant, they were included in the multivariate analysis together, considering their influence on patient prognosis in terms of professional significance. Multivariate Cox regression analyses showed that statistically significant risk factors associated with CSS included age at diagnosis, T-stage, N-stage, bone metastases, brain metastases, liver metastases, lung metastases, chemotherapy, radiotherapy, surgery, and histological grade. For example, patients with higher T-stage or regional lymph node metastasis may have a poorer prognosis. Kaplan-Meier curve analysis visualized the different survival outcomes stratified by each variable included in this study. Log-rank test showed significant differences in CSS between subgroups for T-stage, N-stage, brain metastasis, liver metastasis, lung metastasis, chemotherapy, radiotherapy, surgery, and histological grade (p < 0.05) and no significant differences for age, sex, race, laterality and bone metastasis(p<0.05) (**Figures 2A–N**).

**Table 2:** Univariate and multivariate Cox regression analysis of included variables for CSS in training set.

Characteristics		Univariate analysis				Multivariate analysis		
		HR	95%CI	P-value		HR	95%CI	P-value
Age								
	≤66	Reference				Reference		
	>66	1.144	0.992-1.319	0.065		1.235	1.067-1.430	<0.01
Sex								
	Male	Reference						
	Female	1.096	0.949-1.267	0.208				
Race								
	White	Reference						
	Black	0.939	0.711-1.241	0.659				
	Other	0.904	0.704-1.161	0.430				
Laterality								
	Left	Reference						
	Right	1.006	0.881-1.149	0.931				
T-stage								
	T1	Reference				Reference		
	T2	1.503	1.164-1.942	0.020		1.052	0.808-1.370	0.707
	T3	1.542	1.232-1.930	<0.01		1.251	0.985-1.587	0.066
	T4	2.744	2.099-3.586	<0.01		1.599	1.197-2.137	<0.01
N-stage								
	N0	Reference				Reference		
	N1	1.996	1.717-2.322	<0.01		1.478	1.271-1.739	<0.01
Bone metastases								
	No	Reference				Reference		
	Yes	1.112	0.969-1.277	0.130		1.404	1.185-1.663	<0.01
Brain metastases								
	No	Reference				Reference		
	Yes	2.09	1.737-2.516	<0.01		1.982	1.587-2.476	<0.01
Liver metastases								
	No	Reference				Reference		
	Yes	1.743	1.458-2.084	<0.01		1.673	1.387-2.018	<0.01
Lung metastases								
	No	Reference				Reference		
	Yes	1.296	1.113-1.509	<0.01		1.445	1.205-1.732	<0.01
Chemotherapy								
	No	Reference				Reference		
	Yes	1.179	1.027-1.352	0.019		0.892	0.772-1.031	0.125
Radiotherapy								
	No	Reference				Reference		
	Yes	1.308	1.137-1.505	<0.01		1.203	1.001-1.446	0.049
Surgery								
	No	Reference				Reference		
	Yes	0.359	0.306-0.422	<0.01		0.35	0.291-0.421	<0.01
Histological grade								
Well differentiated Moderately differentiated Poorly differentiated		Reference				Reference		
		1.184	0.999-1.403	0.051		1.376	1.150-1.648	<0.01
		1.763	1.476-2.305	<0.01		2.165	1.771-2.646	<0.01

### Prognostic nomogram construction for CSS

**Figure 3:**
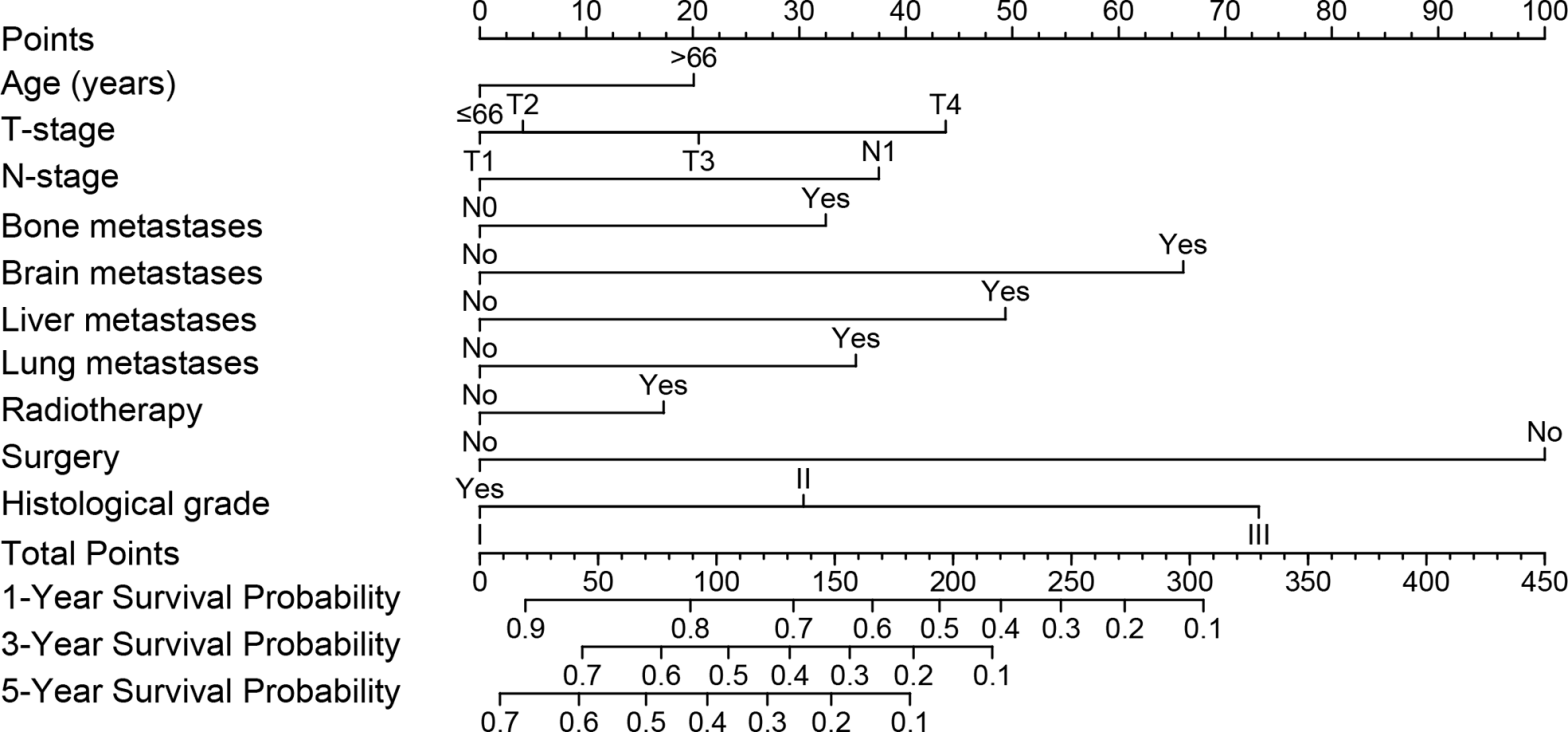
Nomogram model was constructed using the independent prognostic factors predicting the 1-, 3- and 5-year CSS for patients with mccRCC.CSS, Cancer-Specific Survival; mccRCC, metastatic clear cell renal cell carcinoma.

A nomogram was built using the significant prognostic factors for 1-, 3-, and 5-year CSS (**Figure 3**), which was then validated internally using data from the validation set. Each variable in the nomogram was given a corresponding score of 0 to 100 based on the hazard ratio. Each patient could obtain a total score by adding a score in each variable and placed on the total subscale to obtain the probabilities of 1-, 3- and 5-year CSS. For example, age ≤66 years with a score of 0; T4 with a score of 44; N1 with a score of 37; pulmonary metastases with a score of 35; surgical treatment with a score of 0; histological grade of moderately differentiation with a score of 30, the scores corresponding to all variables are added to the total score, which is 146. Then the 1-,3-, and 5-year survival rate is 65%, 35%, and 22%, respectively.

### Calibration and validation of the nomogram

**Figure 4:**
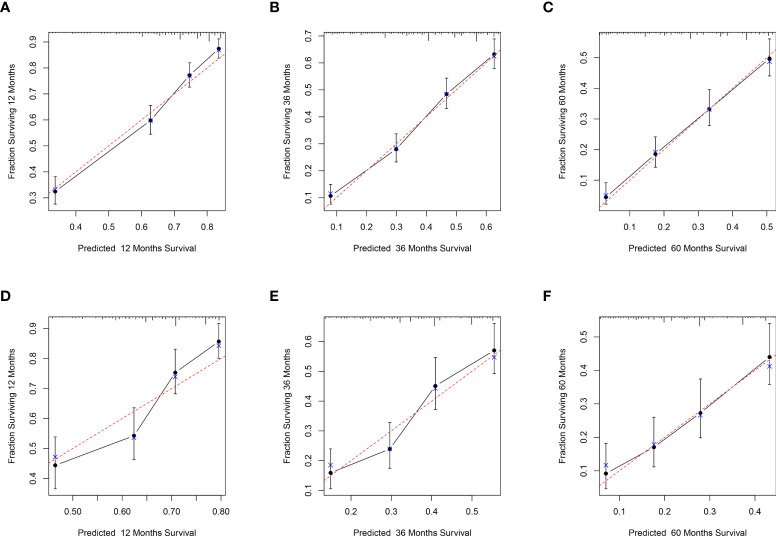
Calibration plots for the nomogram. Calibration plots of 1-year **(A)**, 3-year **(B)**, and 5-year **(C)** CSS in the training set; Calibration plots of 1-year **(D)**, 3-year **(E)**, and 5-year **(F)** CSS in the validation set. CSS, Cancer-Specific Survival.

In the training set, the C-index of the nomogram was 0.707, and in the validation set, the C-index was 0.650, respectively. The data indicated that the nomogram has a good discriminatory ability. Meanwhile, the calibration plots of the nomogram for probabilities of 1-year, 3-year, and 5-year CSS in the training and validation sets displayed consistency between the observed and predicted results (**Figures 4A–F**).

### Comparison of the nomogram and AJCC TNM staging system

**Figure 5:**
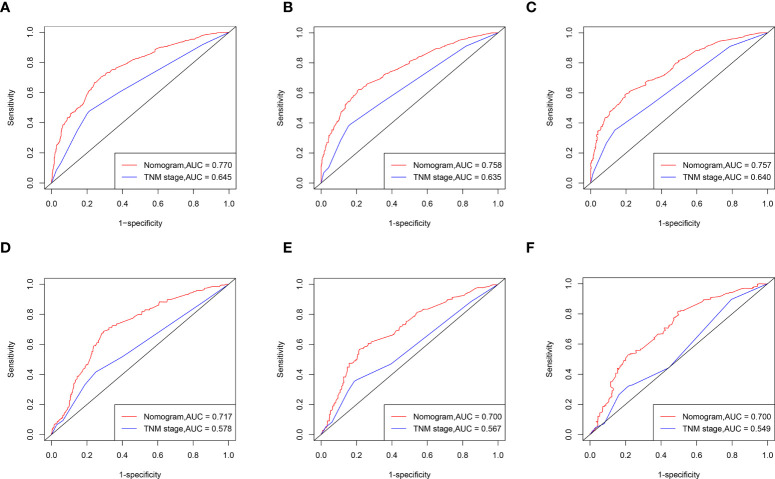
ROC curves of the nomogram for CSS compared with TNM staging. ROC curves comparation of the nomogram and TNM staging for 1-year **(A)**, 3-year **(B)** and 5-year **(C)** CSS in the training set. ROC curves comparation of the nomogram and TNM staging for 1-year **(D)**, 3-year **(E)** and 5-year **(F)** CSS in the validation set. AUC: area under the curve; ROC, receiver operating characteristic; CSS cancer specific survival.

ROC curve analysis showed that the AUC of the 1-, 3-, and 5-year CCS of the nomogram was superior to the TNM staging both in the training and validation sets. (**Figures 5A–F**).

The nomogram performed better than the TNM staging system in the NRI and IDI analyses (**Tables 3**, **4**). In the training set, the 1-, 3-, and 5-year NRI of the nomogram compared to the TNM staging system was 54.5% (95% CI 0.449-0.663), 58.4% (95% CI 0.467-0.701), and 56.3% (95% CI 0.425-0.692), respectively. And the 1-, 3-, and 5-year IDI for the nomogram compared to TNM staging system was 9.0% (p < 0.01), 9.5% (p < 0.01), and 8.2% (p < 0.01), respectively. In the validation set, the 1-, 3-, and 5-year NRI for the nomogram compared to the TNM staging system was 42.5% (95% CI 0.211-0.646), 47.5% (95% CI 0.220-0.626), and 53.8% (95% CI 0.284-0.706), respectively. And the 1-, 3-, and 5-year IDI for the nomogram compared to TNM staging system was 3.0% (p < 0.01), 4.0% (p < 0.01), and 5.7% (p < 0.01).

**Table 3:** NRI of the nomogram in survival prediction for mccRCC patients compared with TNM staging.

NRI (vs.TNM)	Training set	95%CI	Validation set	95%CI
For 1-year CSS (%)	54.5	44.9-66.3	42.5	21.1-64.6
For 3-year CSS (%)	58.4	46.7-70.1	47.5	22.0-62.6
For 5-year CSS (%)	56.3	42.5-69.2	53.8	28.4-70.6

**Table 4:** IDI of the nomogram in survival prediction for mccRCC patients compared with TNM staging.

IDI (vs.TNM)	Training set	p-value	Validation set	p-value
For 1-year CSS (%)	9.0	<0.01	3.0	<0.01
For 3-year CSS (%)	9.5	<0.01	4.0	<0.01
For 5-year CSS (%)	8.2	<0.01	5.7	<0.01

**Figure 6:**
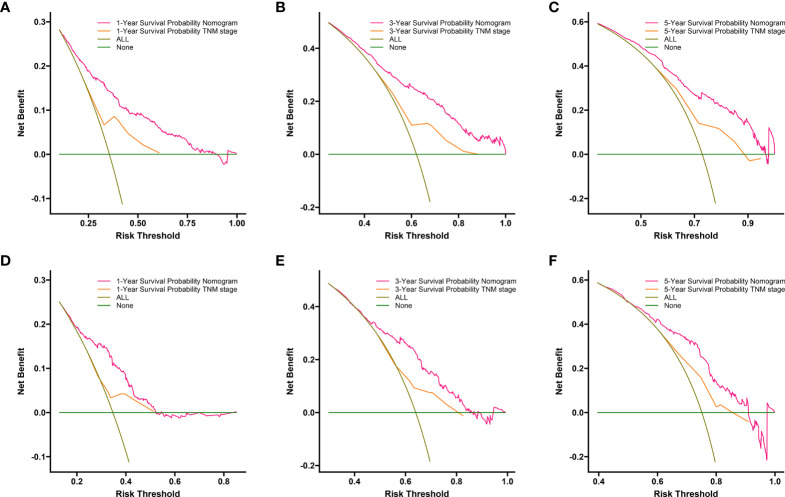
DCA of the nomogram and AJCC TNM staging for 1-year **(A)**, 3-year **(B)** and 5-year **(C)** CSS in training set, and for 1-year **(D)**, 3-year **(E)** and5-year **(F)** CSS in the validation set. The red line represents the nomogram. The orange line represents AJCC TNM stage. CSS, cancer specific survival; DCA, decision curve analyses; AJCC, American Joint Committee on Cancer.

Compared to the AJCC TNM staging system, DCA analysis showed a significant improvement in the net benefit of the nomogram and has a wide range of threshold probabilities both in the training and validation sets (**Figures 6A–F**). This indicated that a new nomogram is more beneficial than the TNM staging system for clinical applications in predicting individual survival outcomes.

### Ability of nomogram to stratify patient risk

**Figure 7:**
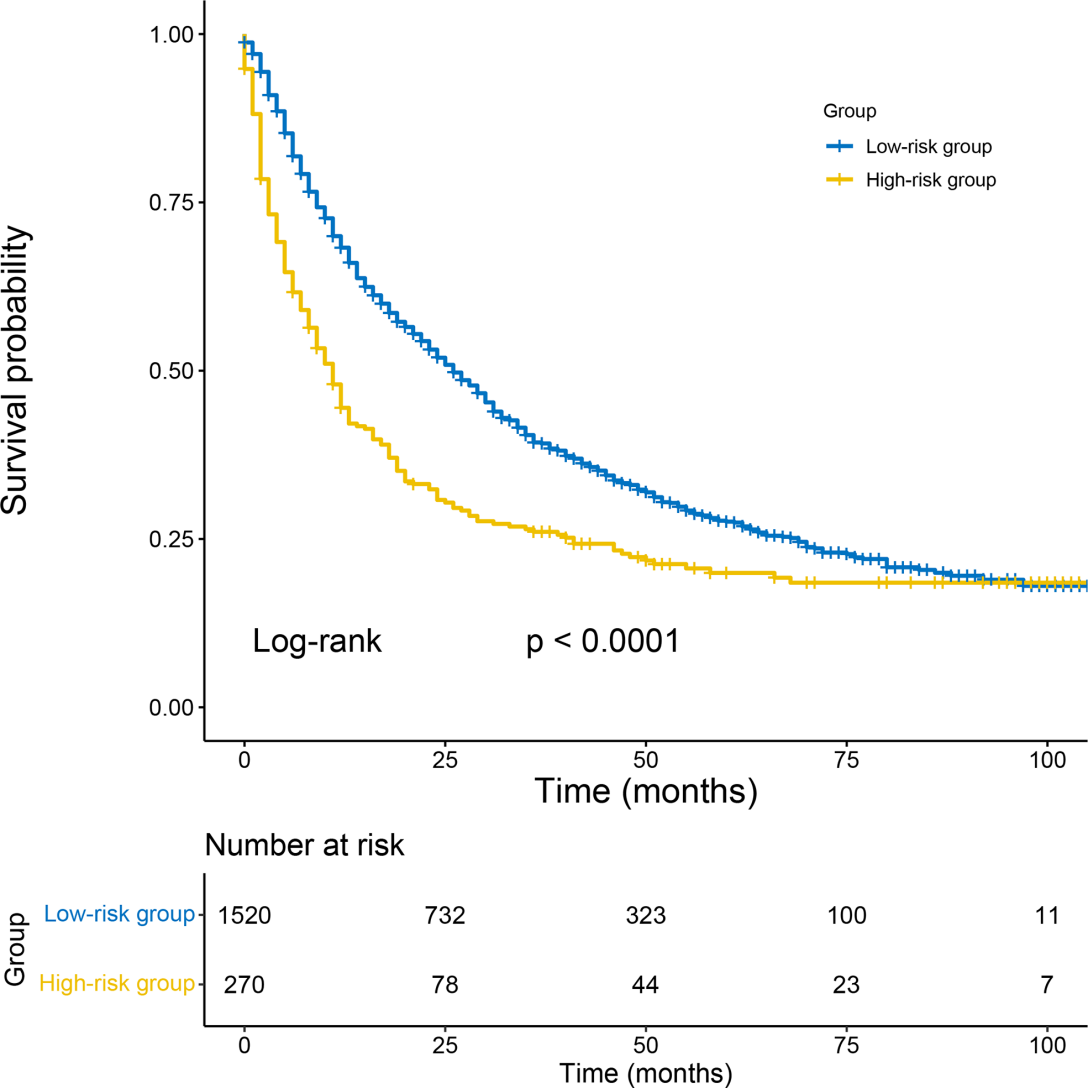
Kaplan–Meier survival analyses to test the risk stratification system within the total cohort. The blue line represents low-risk group, and the yellow line represents high-risk group. Low-risk group (score≤ 257); high-risk group (score >257).

The cut-off value between the high-risk and low-risk groups was determined as 257 by X-tile analysis. The 1790 patients in the total cohort were divided into a high-risk group (total score > 257) and a low-risk group (total score ≤ 257). By Kaplan-Meier analysis, the CSS of 1520 low-risk patients had significantly higher than 270 high-risk patients (p< 0.0001) (**Figure 7**).

### The impact of metastases sites and burden on the outcomes of interest

**Figure 8:**
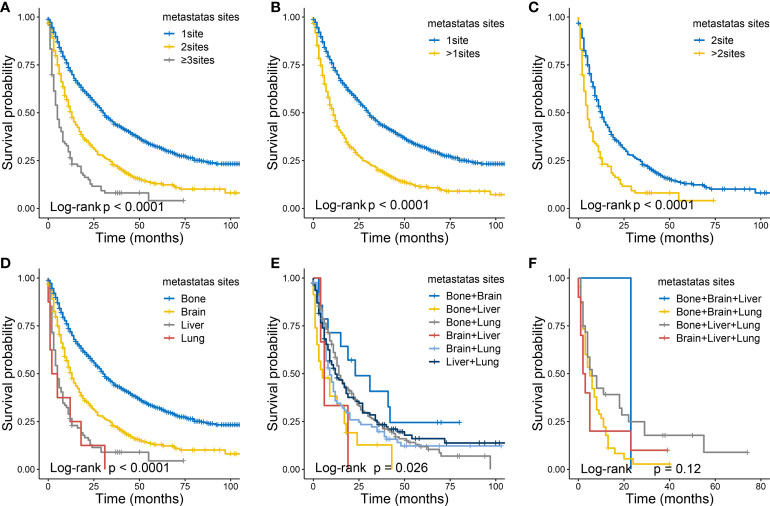
The Kaplan-Meier curves of CSS in ccRCC patients according to metastatic status: 1 site versus 2 sites verses ≥3 sites **(A)**, 1 site versus >1 sites **(B)**, 2 sites versus >2 sites **(C)**. The Kaplan-Meier curves of CSS in ccRCC patients according to metastatic status: with single site **(D)**, with two sites **(E)** , with three sites **(F)**.

As shown in **Figure 8**, in terms of the CSS, as the number of metastases increased in patients with distant metastasis, the long-term CSS probabilities decreased significantly (**Figure 8A**). Besides, patients with multiple metastatic sites had worse CSS than those with single metastatic site (**Figure 8B**), and the same in the comparisons between two metastatic sites and more (**Figure 8C**). Among all metastatic sites, those with single liver or lung liver metastasis had the worst CSS (**Figure 8D**). Moreover, survival curves of patients with two metastatic sites are shown those with brain + liver or bone + liver metastasis had the worst CSS (**Figure 8E**). And no significant differences were identified in CSS (**Figure 8F**) for various metastatic patterns among patients with three metastatic sites.

## Discussion

The three common pathological types of RCC are clear cell carcinoma, chromophobe cell carcinoma, and papillary cell carcinoma. The ccRCC is most prone to distant metastasis (7, 8), and distant metastasis is considered to be an important factor in poor prognosis of patients with ccRCC, with a median survival time of fewer than 2 years (6). Riccardo Campi’s study has also indicated ccRCC is with worse prognosis and a higher risk of relapse (19). However, most patients with mccRCC can benefit from cytoreductive nephrectomy (CN). In addition, with the development of treatment methods for patients with mRCC, many new treatment methods have emerged, such as targeted therapy and immunotherapy (20). This enables patients with mRCC to obtain a better prognosis. The TNM staging system of AJCC is the most commonly used prognostic evaluation system for RCC (12). However, in clinical practice, significant survival differences were observed in patients with mccRCC with the same TNM stage. Therefore, it is necessary to establish an accurate and appropriate survival prediction model for patients with mccRCC. In this study, a large number of data were collected from the SEER database to establish and validate the survival and prognosis nomogram of patients with mccRCC. This study is the first time to use the SEER database to establish a survival and prognosis prediction model for patients with mccRCC and compared the nomogram with the TNM staging system using the NRI and IDI, aiming to better predict the survival and prognosis of patients at the population level.

This study comprehensively explored the impact of relevant factors in the SEER database on the survival of patients with mccRCC. At the same time, we combined the variables screened by a multivariable Cox regression model, and constructed and internally validated a relatively accurate nomogram to predict the CSS of patients with mccRCC. This approach produces a relatively simple and accurate tool that contains only important variables related to survival outcomes without sacrificing accuracy. The final survival nomogram produced good accuracy of survival prognosis, which far exceeded the traditional TNM staging model (21). In addition, different risk groups can be constructed according to the nomogram score and patient individualized consultation and follow-up arrangements can be tailed for different risk groups (22).

In recent years, nomograms have been widely used. Martini’s nomogram applied to the prediction of significant renal function decline after open, laparoscopic, and robotic partial nephrectomy and the result show that the robot-assisted partial nephrectomy have excellent oncologic and functional outcomes (23). Moreover, martini’s nomogram is a valid tool for predicting the decline in renal function at 6 and 12 months after robot-assisted partial nephrectomy and laparoscopic partial nephrectomy (24). The nomograms developed using the SEER database have been widely used to predict the prognosis of various malignant tumors, such as Ewing sarcoma, penile cancer, and cardiac sarcoma (25). Our current study constructs an internally validated nomogram for patients with mccRCC for the first time. It can intuitively and effectively predict the survival and prognosis of patients. The variables in the nomogram are independent factors affecting CSS, which can better predict the survival rate of patients with mccRCC. Using this nomogram, we will be able to more accurately predict the future survival rate of patients. Although the C-index and AUC of nomograms in the training set and validation set are not high enough, the prediction ability of the model is more accurate than using the current TNM staging to predict the prognosis. In addition to C-index and AUC, NRI and IDI are also used to compare the prediction ability of nomogram and TNM staging system, which is specially used to compare the prediction ability of old and new models. Further analysis of DCA proved that it was significantly superior to the TNM staging system in clinical application. The risk stratification model based on this nomogram can effectively divide the patients in the total cohort into two risk groups (high-risk group and low-risk group), and can distinguish CSS. The results of this study may be particularly helpful in predicting postoperative survival in patients with mccRCC.

The nomogram for predicting the prognosis of patients with mccRCC has ten prognostic factors, including diagnostic age, T-stage, N-stage, bone metastases, brain metastases, liver metastases, lung metastases, radiotherapy, surgery, and histological grade. In terms of age, several studies have shown that the prognosis of elderly patients is worse than that of young patients (26, 27). This may be related to the higher resistance of young people. Lymph node metastasis means a poor prognosis. Previous studies have shown that lymph node metastasis is associated with reduced survival in patients with locally advanced RCC (28). In a recent study, Yu and his colleagues found that the prognosis of patients with lymph node involvement was significantly worse than the patients without lymph node involvement (29). Other studies have found that the 10-year overall survival (OS) of patients with RCC lymph node metastasis is between 15% and 26% (30, 31). Distant metastasis indicates a poor prognosis. Previous studies have pointed out that lung metastasis is not an independent risk factor for the prognosis of RCC patients, but the inclusion of a large number of patients with multiple distant metastases will also affect the prognosis of patients to a certain extent (32, 33). Other studies (34) found that compared with RCC patients with lung metastasis alone, patients with bone, brain, and liver metastasis had an increased risk of disease-specific death, which were 1.524, 1.664, and 1.355 times respectively. Patients with distant metastases can benefit from CN. Pooja Ghatalia (35) reported in the study of Urology in the United States that CN benefits significantly in patients with mRCC. In addition, compared with systemic treatment alone, patients receiving CN and systemic treatment have greater survival benefits. Jack R Andrews (36) found that after RCC patients received CN, regardless of whether they received systemic treatment or not, about half of the patients may avoid systemic treatment within 1 year in the long-term follow-up, and some of the metastases achieved long-term survival without systemic treatment. Therefore, CN is an appropriate and meaningful treatment for patients with mccRCC. Radiotherapy is a risk factor for patients with mccRCC, which may be related to the side effects of radiotherapy. It reminds us that patients with mccRCC should avoid radiotherapy as much as possible.

In the past 20 years, with our further understanding of the molecular drivers of RCC, significant progress has been made in the targeted therapy and immunotherapy of metastatic renal carcinoma. The research mainly focus on the histology of clear cell RCC (ccRCC), which accounts for more than 75% of all cases (37). Within ccRCC, the von Hippel Lindau (VHL) and hypoxia-induced factor (HIF) pathways have been identified as important drivers of pathogenesis. Perhaps the most notable among these is sunitinib, a first-in-class tyrosine kinase inhibitor of VEGF receptor, which demonstrated its superiority over interferon alpha in overall survival (OS), progression-free survival (PFS), and response rate (RR) in a landmark trial published in 2007. Sunitinib has since become the standard-of-care comparator for clinical trials in RCC. The mechanistic target of rapamycin (mTOR) inhibitors temsirolimus and everolimus have also been shown to be clinically effective and are approved as largely second-line agents (20). The effect of targeted therapy is useful, but there are also side effects. This nomogram evaluates the CSS of patients for 1, 3 and 5 years. If the CSS of patients is too low, taking into account the side effects of targeted therapy and the poor physical condition of patients, targeted therapy may not be considered. In addition, patients identified as high-risk group through nomogram may also consider not to carry out targeted treatment if their physical condition is poor. The practical role of this nomogram in targeted therapy needs further prospective research.

As for immunotherapy, modern immune checkpoint inhibitors (ICI) targeting programmed cell death 1 (PD-1),programmed cell death ligand 1 (PD-L1) and cytotoxic T lymphocyte-associated antigen 4 (CTLA-4) have renewed the promise of immunotherapy in the treatment of RCC and show potential for achieving durable remission. PD-1 and CTLA-4 are both expressed on activated T cells and act to down-regulate T cell response when in contact with their ligands (20).Cancer cells have leveraged this mechanism to evade immune surveillance by expressing PD-L1 (38). Indeed, PD-L1 can be significantly overexpressed in RCC (39). The treatments acting on this pathway include the PD-1 inhibitors nivolumab and pembrolizumab, the PD-L1 inhibitors avelumab and atezolizumab, and the CTLA-4 inhibitor ipilimumab. The nomogram in our study can be used to determine whether to receive immunotherapy according to the survival rate and risk stratification predicted by the nomogram, and observe the effect of immunotherapy with different survival rates and risk stratification

There are several significant advantages to note in this study. First, to our knowledge, this is the first study to predict CSS by prognostic nomogram in patients with mccRCC. Then, the number of patients in this study was relatively large enough to construct a prognostic nomogram with good performance (n = 1790). In addition, the variables in the nomogram are easy to obtain in most hospitals, and have good applicability in our nomogram. Finally, the ROC curve, DCA, NRI and IDI analysis of this study shows that the nomogram can more accurately predict the CSS of patients with mccRCC and has clinical applicability. These findings are consistent with the internal validation results of nomogram prediction. The prediction ability of this model is more accurate than using the current TNM stage system to predict the prognosis. At the same time, we divided the study population into two risk groups according to the prognostic nomogram, which makes it easier to find patients with poor survival results.

However, there are some limitations in this study. First, this is a retrospective study based on the SEER database, which means that the results of this study are inevitably affected by selection bias. In addition, we excluded patients with unknown variable information, which is also an important source of selection bias. Secondly, the SEER database has some limitations. For example, the SEER database lacks some factors that are also important for the prognosis of patients with mccRCC, such as laboratory results and new therapies such as targeted therapy. Finally, although internal validation was performed in the validation set, the results of this validation method were not perfect because the patients in the training and validation sets were from the same database. Therefore, large prospective clinical trials are needed for external validation.

## Conclusion

In this study, we developed and validated a nomogram to predict the CSS rate in mccRCC patients, which showed consistent reliability and clinical applicability. Nomogram may assist clinicians in evaluating the risk factors of patients and formulating an optimal individualized treatment strategy. However, further evaluation of other patient groups is needed to determine the external validity of our findings.

## Data availability statement

The datasets presented in this study can be found in online repositories. The names of the repository/repositories and accession number(s) can be found in the article/**Supplementary Material**.

## Ethics statement

The studies involving human participants were reviewed and approved by Medical Ethics Committee of Jinan University’s First Affiliated Hospital. Written informed consent for participation was not required for this study in accordance with the national legislation and the institutional requirements.

## Author contributions

GH, JL, and SC conceived and designed the study. YZ, LiaW, and ZG provided administrative support. GH, JL, ZC, XQ, LiwW, and JL collected and assembled the data. LB, JR, WZ, YL, and YyL contributed to data processing, GH, JL, KD, and XL contributed to processing pictures, interpretation of results, and drafting. All authors read and approved the manuscript.

## Funding

This study was supported by Science and Technology Planning for Fundamental and Applied Basic Research of Guangzhou-Municipal School (Institution) Joint Funding(202201020037, 202201020051), Clinical Frontier Technology Program of the First Affiliated Hospital of Jinan University, China (No. JNU1AF-CFTP- 2022-a01207), Science Research Project of Talent Introduction Fund of the First Affiliated Hospital, Jinan University(808037), and The Medical Scientific Research Foundation of Guangdong Province (A2019221).

## Acknowledgments

The authors are grateful to Prof. Jun Lyu’s team at the Department of Clinical Research, The First Hospital of Jinan University for their guidance on the methodology of this study.

## Conflict of interest

The authors declare that the research was conducted in the absence of any commercial or financial relationships that could be construed as a potential conflict of interest.

## Publisher’s note

All claims expressed in this article are solely those of the authors and do not necessarily represent those of their affiliated organizations, or those of the publisher, the editors and the reviewers. Any product that may be evaluated in this article, or claim that may be made by its manufacturer, is not guaranteed or endorsed by the publisher.
